# Cardiac magnetic resonance imaging demonstrates biatrial stunning after catheter ablation of persistent atrial fibrillation

**DOI:** 10.1186/1532-429X-14-S1-P203

**Published:** 2012-02-01

**Authors:** Kai Muellerleile, Michael Groth, Daniel Steven, Boris Hoffmann, Jakob Lueker, Arian Sultan, Ulf K Radunski, Gunnar Lund, Gerhard Adam, Thomas Rostock, Stephan Willems

**Affiliations:** 1 Center for Cardiology and Cardiovascular Surgery, University Heart Center Hamburg, Hamburg, Germany; 2 Department of Diagnostic and Interventional Radiology, University Medical Center Hamburg-Eppendorf, Hamburg, Germany

## Summary

This study evaluated the course of active left atrial (LA), LA appendage (LAA) and right atrial (RA) emptying with respect to ablation-related inflammation after persistent AF ablation by cardiac magnetic resonance imaging (CMR).

## Background

Catheter ablation of persistent atrial fibrillation (AF) aims at reestablishing active atrial emptying. However, little is known on recovery of active atrial emptying and inflammation after successful ablation of persistent AF.

## Methods

CMR was performed in 19 consecutive patients at baseline (BL) within 24 hours and at follow-up (FU) 5±2 months after restoration of sinus rhythm by catheter ablation of persistent AF. Velocity encoded (VENC) MRI was used to assess active LA, LAA and RA emptying: Active LA and RA emptying were quantified by calculating the active emptying fraction (AEF) from transmitral VENC-MRI flow profiles. LAA function was quantified by measurements of peak a-wave velocities from VENC-MRI flow profiles perpendicular to the LAA orifice. Peri-atrial inflammation was assessed using edema-sensitive black-blood T2-weighted MRI.

## Results

There was a significant increase in LA-AEF from 17 (12-26) % at baseline to 25 (23-36) % at follow-up (p<0.01). A significant increase was also found for LAA a-wave velocities from 45 (30-67) cm/s at baseline to 62 (47-72) cm/s at follow-up (p<0.01). Furthermore, RA-AEF significantly improved from 32 (18-36) % at baseline to 40 (37-50) % at follow-up (p<0.0001). Conversely, the area of peri-atrial edema on T2-weighted MRI decreased from 2340 (1944-2745) mm^2^ at baseline to 423 (237-823) mm^2^ at follow-up (p<0.0001).

## Conclusions

Catheter ablation of persistent AF results in inflammation-related biatrial stunning. Disappearance of inflammation is associated with recovery of active LA, LAA and RA emptying.

## Funding

No external funding.

